# Association between triglyceride glucose- systemic immune inflammation index and deep venous thrombosis in fresh fracture patient: a real-world retrospective study

**DOI:** 10.3389/fendo.2026.1700809

**Published:** 2026-03-12

**Authors:** He Chen, Ke Chen, Xuewen Xie, Mei Lin, Weidan Yuan, Shiyun Luo

**Affiliations:** The Eighth Clinical Medical College of Guangzhou University of Chinese Medicine/Foshan Hospital of Traditional Chinese Medicine, Foshan, China

**Keywords:** deep venous thrombosis (DVT), fracture, K-means clustering, real-world retrospective study, triglyceride glucose-systemic immune inflammation index (TyG-SII)

## Abstract

**Background:**

Currently, systemic inflammation indices and insulin resistance are both recognised as high-risk factors for venous thromboembolic diseases. However, there is a lack of research on the relationship between the triglyceride-glucose-systemic inflammation index (TyG-SII) and the risk of lower limb deep vein thrombosis (DVT) in populations with traumatic fractures. This study aims to investigate the relationship between TyG-SII and the risk of DVT.

**Methods:**

The study participants were inpatients from the Orthopaedic Centre of Foshan Traditional Chinese Medicine Hospital. Participants were divided into three groups using K-means clustering analysis based on changes in TyG-SII. Multivariate binary logistic regression analysis was used to explore the association between different groups (based on different levels of TyG-SII and DVT. Restricted cubic spline (RCS) regression models were used to explore the potential nonlinear association between TyG-SII and DVT events. Receiver operating characteristic (ROC) curves were employed to quantify the predictive ability of TyG-SII for DVT. Subgroup analyses further confirmed the relationship between TyG-SII and DVT in different populations.

**Results:**

Among the 5,455 patients, 1,991 (36.50%) developed DVT, and 646 (11.84%) developed only MCVT. After adjusting for various potential confounding factors, patients with moderate (OR = 0.86, 95% CI: 0.66–0.98) and high levels of TyG-SII had a significantly lower risk of DVT compared to those with low levels of TyG-SII (OR = 0.73, 95% CI: 0.58–0.95). Compared with the lowest quartile (Q1) of baseline TyG-SII, the third quartile (Q3) showed the most significant protective effect (OR = 0.46, 95% CI: 0.39–0.55). Additionally, there was a nonlinear U-shaped relationship between baseline TyG-SII and DVT risk, with thresholds of 242.14 and 3710.98, respectively.

**Conclusions:**

For patients with traumatic fractures, TyG-SII is independently associated with the risk of lower limb DVT. Maintaining a good TyG-SII index helps prevent the occurrence of DVT after traumatic fractures.

## Introduction

Deep vein thrombosis (DVT) is a venous return disorder caused by abnormal blood clotting in deep veins, commonly occurring in the lower limbs of patients with traumatic fractures. When a DVT clot detaches, it may cause pulmonary embolism (PE), which can be sudden and fatal. Therefore, DVT and PE are collectively referred to as venous thromboembolism (VTE). Currently, there are approximately 10 million new cases of VTE worldwide each year, making it the third most common vascular disease (1, 2). Although the incidence of DVT reported in Asian countries is significantly lower than in North American countries, approximately one-third to one-fifth of the incidence in Western countries (3), the incidence of DVT in patients with traumatic fractures continues to rise. Epidemiological data show that the overall incidence of DVT in patients undergoing orthopaedic surgery ranges from 9.1% to 11.1% (4). The situation is even more severe in specific high-risk groups: the incidence of DVT in patients with hip fractures during the perioperative period ranges from 16.6% to 34.98% (5). Notably, in elderly patients with hip fractures, the incidence of DVT without preoperative intervention can rise as high as 42% to 50% (6, 7). The incidence of DVT after surgery for traumatic spinal fractures also reaches as high as 52.7% (8). With the continuous increase in the global volume of trauma and orthopaedic surgeries, DVT has become a significant clinical challenge threatening patients’ quality of life and safety. This highlights the urgency of initiating early identification, strengthening prevention, and reducing the incidence of DVT in trauma fracture patients from the moment of admission.

Currently, multiple risk factors are known to be associated with DVT, but relying solely on risk scores is insufficient for timely warning or accurate prediction of DVT incidence risk. Therefore, in clinical practice, blood biomarkers are often combined to assess individuals at high risk of DVT, such as D-dimer, platelets, and fibrinogen (9). However, these markers lack specificity and are easily influenced by various pathophysiological states, limiting their value in trauma fracture patients. Recent studies have focused on identifying new markers that balance sensitivity and specificity and complement blood biomarkers. Increasing evidence suggests that immune-inflammatory response-related indices play a key role in the development of deep vein thrombosis in trauma fracture patients during the perioperative period (10, 11). Among these, the systemic immune-inflammatory index (SII) demonstrates higher sensitivity and specificity for deep vein thrombosis patients compared to other inflammatory indices such as NLR, PLR, and SIRI (12–14). Additionally, an increasing number of researchers have recognised the key non-inflammatory mechanism in the pathogenesis of DVT and VTE—metabolic disorders (15). Insulin resistance (IR) is characterised by reduced physiological responsiveness of peripheral tissues to insulin and is the core pathological mechanism of various metabolic disorders. Serum triglyceride-glucose product (TyG) serves as a new indicator of IR, characterised by its simplicity, ease of acquisition, and high specificity (16). Previous studies have shown that IR leads to vascular damage, thrombosis, and atherosclerotic plaque rupture in cardiovascular diseases and is closely associated with lower extremity venous thrombosis (17, 18). A recent study on predictive models for intracerebral haemorrhage patients explicitly stated that the TyG index can serve as one of the predictive factors for lower limb venous thrombosis (19). However, studies on the risk of DVT in trauma fracture patients using the TyG index alone remain limited.

To address the lack of metabolic disorder mechanisms in the field of DVT risk in patients with traumatic fractures, and to further consider the immune-inflammatory response coagulation mechanism, we hypothesised that the TyG-SII index may better predict DVT progression and aimed to investigate their association from real-world clinical data.

## Methods

### Research subjects and data collection

This retrospective study was conducted in accordance with the Declaration of Helsinki. The research protocol was reviewed and approved by the Ethics Committee of Foshan Traditional Chinese Medicine Hospital. Given the retrospective design and the use of anonymized clinical data, the requirement for obtaining written informed consent from patients or their guardians was waived by the Ethics Committee.

We reviewed trauma patients admitted to the 15 orthopaedic subspecialty departments of the Orthopaedic Centre at Foshan Municipal Hospital from January 2019 to January 2024 through the hospital’s Computer Information Data Centre. Patients were initially identified by screening for diagnoses containing the term ‘fracture’ and further excluded those with diagnoses including ‘old fractures,’ ‘pathological fractures,’ ‘open fractures,’ ‘postoperative infection after fracture surgery,’ ‘non-union after fracture surgery,’ and ‘spinal fractures,’ resulting in an initial extraction of 30,605 patients from the data centre. Data were obtained from the electronic medical record system, clinical laboratory system, and imaging system using hospital ID numbers and patient names, including demographic information (age, gender, weight, height, alcohol consumption, fasting blood glucose upon admission, blood pressure upon admission), past medical history (hypertension, hyperlipidaemia, diabetes, heart disease, stroke, chronic respiratory diseases, digestive system diseases, liver diseases, kidney diseases), fracture-related data (fracture location, time from admission to vascular ultrasound examination, fractured limb, vascular ultrasound results), Admission laboratory biomarker results, including complete blood count (white blood cells, red blood cells, neutrophils, lymphocytes, haemoglobin, platelets, etc.), inpatient biochemistry + ions (alanine aminotransferase, aspartate aminotransferase, alkaline phosphatase, triglycerides, total cholesterol, creatinine, uric acid, sodium ions, chloride ions, potassium ions, etc.), and coagulation panel (D-dimer, antithrombin III, thrombin time, etc.). More specific indicators refer to **Table 1**.

**Table 1:** Baseline characteristics of participants by K-means clustering analysis.

Characteristic	Overall (N=5455)	Change in the TyG-SII			P value
		Cluster 1 (n=2795)	Cluster 2 (n=1383)	Cluster 3 (n=1276)	
Age (years)	48.08 ± 15.55	46.92 ± 16.18	49.41 ± 14.45	49.20 ± 15.10	<0.001
Times (days)	4.47 ± 2.23	4.48 ± 2.25	4.48 ± 2.22	4.42 ± 2.21	0.669
FBG (mmol/L)	6.00 ± 1.90	5.70 ± 1.51	6.51 ± 2.55	6.10 ± 1.74	<0.001
Sbp (mmHg)	130.50 ± 22.07	130.28 ± 22.38	130.42 ± 21.26	131.06 ± 22.27	0.572
Dbp (mmHg)	76.06 ± 12.52	76.01 ± 12.61	75.97 ± 12.41	76.25 ± 12.43	0.808
K (mmol/L)	3.86 ± 0.35	3.85 ± 0.34	3.88 ± 0.35	3.87 ± 0.36	0.035
Cl (mmol/L)	102.78 ± 2.90	102.97 ± 2.92	102.67 ± 2.82	102.48 ± 2.90	<0.001
LDH (U/L)	198.07 ± 66.56	195.59 ± 65.25	198.04 ± 68.15	203.54 ± 67.39	0.002
RBP (mg/L)	39.62 ± 13.78	38.93 ± 13.79	40.86 ± 13.61	39.79 ± 13.85	<0.001
TCH (mmol/L)	4.70 ± 1.12	4.58 ± 1.08	4.94 ± 1.17	4.71 ± 1.10	<0.001
TG (mmol/L)	1.35 ± 0.95	1.11 ± 0.59	1.91 ± 1.39	1.28 ± 0.71	<0.001
TP (g/L)	68.26 ± 6.11	68.15 ± 5.93	68.65 ± 6.36	68.07 ± 6.22	0.023
Ca (mmol/L)	2.30 ± 0.12	2.30 ± 0.12	2.31 ± 0.12	2.30 ± 0.12	0.089
P (mmol/L)	1.13 ± 0.23	1.14 ± 0.23	1.14 ± 0.23	1.12 ± 0.22	0.026
CRP (mg/L)	30.45 ± 33.04	28.51 ± 31.04	30.73 ± 32.89	34.38 ± 36.89	<0.001
DBIL (umol/L)	4.09 ± 2.23	4.18 ± 2.16	3.81 ± 2.21	4.20 ± 2.39	<0.001
TBIL (umol/L)	15.29 ± 7.59	15.28 ± 6.85	14.87 ± 8.49	15.75 ± 8.06	0.011
ASHO (IU/ml)	63.81 ± 69.30	66.71 ± 74.67	60.85 ± 61.38	60.65 ± 64.79	0.006
HDL-C (mmol/L)	1.29 ± 0.24	1.31 ± 0.23	1.23 ± 0.23	1.29 ± 0.25	<0.001
LDL-C (mmol/L)	2.85 ± 0.87	2.76 ± 0.85	3.02 ± 0.90	2.87 ± 0.86	<0.001
APOB (g/L)	1.11 ± 0.33	1.06 ± 0.32	1.19 ± 0.32	1.10 ± 0.33	<0.001
ALT (U/L)	30.02 ± 34.80	28.42 ± 32.98	32.22 ± 37.51	31.13 ± 35.48	0.002
AST (U/L)	29.11 ± 28.52	28.02 ± 26.28	30.14 ± 32.45	30.40 ± 28.59	0.014
ALP (U/L)	78.98 ± 32.63	77.91 ± 33.55	81.09 ± 32.99	79.03 ± 29.99	0.012
CHE (U/mL)	923.81 ± 2554.82	879.19 ± 2448.51	1134.96 ± 2906.02	798.13 ± 2365.74	0.002
Cr (umol/L)	70.88 ± 33.03	70.69 ± 34.50	70.70 ± 20.53	71.48 ± 40.00	0.761
UA (umol/L)	322.73 ± 104.23	316.42 ± 99.80	340.92 ± 110.05	316.84 ± 105.01	<0.001
PA (mg/L)	234.00 ± 71.02	228.07 ± 69.65	246.99 ± 74.02	232.90 ± 68.84	<0.001
Na (mmol/L)	140.61 ± 2.67	140.69 ± 2.64	140.77 ± 2.62	140.27 ± 2.76	<0.001
Mg (mmol/L)	0.90 ± 0.08	0.90 ± 0.07	0.90 ± 0.08	0.90 ± 0.08	0.515
GLO (g/L)	28.91 ± 4.21	28.78 ± 4.11	29.18 ± 4.40	28.89 ± 4.22	0.017
UREA (mmol/L)	5.14 ± 1.82	5.06 ± 1.84	5.22 ± 1.72	5.23 ± 1.88	0.004
MONO#(X10^9/L)	0.65 ± 0.27	0.62 ± 0.25	0.63 ± 0.26	0.71 ± 0.28	<0.001
BASO#(X10^9/L)	0.03 ± 0.02	0.03 ± 0.02	0.03 ± 0.02	0.03 ± 0.02	<0.001
NEUT%	67.20 ± 9.90	65.06 ± 9.83	65.97 ± 8.99	73.21 ± 8.47	<0.001
LYMH%	22.54 ± 8.46	24.38 ± 8.48	23.66 ± 7.65	17.31 ± 6.97	<0.001
MONO%	7.51 ± 1.96	7.62 ± 1.89	7.44 ± 1.97	7.35 ± 2.08	<0.001
EOS%	2.37 ± 2.39	2.54 ± 2.43	2.55 ± 2.40	1.82 ± 2.20	<0.001
MPV (fL)	9.57 ± 1.12	9.64 ± 1.13	9.60 ± 1.16	9.39 ± 1.02	<0.001
WBC(X10^9/L)	8.59 ± 2.67	8.15 ± 2.56	8.50 ± 2.61	9.67 ± 2.67	<0.001
RBC(X10^12/L)	4.27 ± 0.79	4.30 ± 0.75	4.31 ± 0.83	4.18 ± 0.80	<0.001
PLT(X10^9/L)	245.18 ± 78.55	237.03 ± 75.91	246.03 ± 79.26	262.11 ± 80.76	<0.001
EOS#(X10^9/L)	0.19 ± 0.19	0.19 ± 0.18	0.20 ± 0.20	0.16 ± 0.18	<0.001
BASO%	0.38 ± 0.26	0.40 ± 0.27	0.39 ± 0.25	0.31 ± 0.22	<0.001
NEUT#(X10^9/L)	5.90 ± 2.37	5.43 ± 2.24	5.70 ± 2.18	7.17 ± 2.37	<0.001
LYMH#(X10^9/L)	1.83 ± 0.65	1.88 ± 0.63	1.93 ± 0.67	1.60 ± 0.60	<0.001
HGB (g/L)	121.47 ± 21.10	122.47 ± 20.53	122.48 ± 21.96	118.18 ± 21.06	<0.001
P-LCR(%)	23.09 ± 7.93	23.57 ± 8.09	23.36 ± 8.14	21.74 ± 7.16	<0.001
Height (cm)	158.40 ± 8.75	158.37 ± 8.57	158.36 ± 9.01	158.49 ± 8.84	0.904
Weight (kg)	58.26 ± 11.46	58.32 ± 11.69	58.12 ± 11.47	58.30 ± 10.94	0.862
BMI (kg/m²)	23.16 ± 3.95	23.20 ± 4.12	23.11 ± 3.84	23.15 ± 3.68	0.780
TYG	1.22 ± 0.60	1.01 ± 0.51	1.62 ± 0.63	1.22 ± 0.51	<0.001
SII	876.75 ± 619.45	755.59 ± 619.14	779.79 ± 522.94	1247.33 ± 569.95	<0.001
TYG-SII	1060.09 ± 924.73	778.47 ± 882.16	1222.91 ± 861.88	1500.69 ± 865.95	<0.001
D-Dimer(ug/ml)	5.99 ± 9.26	6.09 ± 9.85	5.53 ± 8.36	6.25 ± 8.86	0.090
Sex					<0.001
Female	2343 (42.95%)	1220 (43.63%)	587 (42.44%)	536 (42.01%)	
Male	3112 (57.05%)	1576 (56.37%)	796 (57.56%)	740 (57.99%)	
FL					0.002
right	2382 (43.67%)	1275 (45.60%)	552 (39.91%)	555 (43.50%)	
left	3073 (56.33%)	1521 (54.40%)	831 (60.09%)	721 (56.50%)	
MCVT					0.041
No	4809 (88.16%)	2435 (87.09%)	1232 (89.08%)	1142 (89.50%)	
Yes	646 (11.84%)	361 (12.91%)	151 (10.92%)	134 (10.50%)	
DVT					0.003
No	3464 (63.50%)	1727 (61.77%)	878 (63.49%)	859 (67.32%)	
Yes	1991 (36.50%)	1069 (38.23%)	505 (36.51%)	417 (32.68%)	
Drink					<0.001
No	2203 (40.38%)	1110 (39.70%)	555 (40.13%)	538 (42.16%)	
Yes	3252 (59.62%)	1686 (60.30%)	828 (59.87%)	738 (57.84%)	
Hypertension					<0.001
No	4165 (76.35%)	2160 (77.25%)	1062 (76.79%)	943 (73.90%)	
Yes	1290 (23.65%)	636 (22.75%)	321 (23.21%)	333 (26.10%)	
Hyperlipidaemia					<0.001
No	4975 (91.20%)	2543 (92.57%)	1270 (93.31%)	1162 (93.33%)	
Yes	378 (6.93%)	204 (7.43%)	91 (6.69%)	83 (6.67%)	
Diabetes					<0.001
No	5165 (94.68%)	2645 (95.18%)	1318 (95.92%)	1202 (95.09%)	
Yes	252 (4.62%)	134 (4.82%)	56 (4.08%)	62 (4.91%)	
Cancer					0.672
No	5396 (98.92%)	2766 (99.32%)	1366 (99.27%)	1264 (99.53%)	
Yes	35 (0.64%)	19 (0.68%)	10 (0.73%)	6 (0.47%)	
CRD					0.977
No	4909 (89.99%)	2518 (90.74%)	1243 (90.73%)	1148 (90.54%)	
Yes	504 (9.24%)	257 (9.26%)	127 (9.27%)	120 (9.46%)	
Asthma					0.364
No	5256 (96.35%)	2697 (96.60%)	1325 (96.01%)	1234 (97.01%)	
Yes	188 (3.45%)	95 (3.40%)	55 (3.99%)	38 (2.99%)	
LD					0.583
No	5210 (95.51%)	2670 (95.97%)	1316 (95.78%)	1224 (96.53%)	
Yes	214 (3.92%)	112 (4.03%)	58 (4.22%)	44 (3.47%)	
Stroke					0.696
No	5353 (98.13%)	2742 (98.21%)	1359 (98.55%)	1252 (98.43%)	
Yes	90 (1.65%)	50 (1.79%)	20 (1.45%)	20 (1.57%)	
HD					0.564
No	4922 (90.23%)	2536 (91.06%)	1242 (90.39%)	1144 (90.08%)	
Yes	507 (9.29%)	249 (8.94%)	132 (9.61%)	126 (9.92%)	
KD					0.236
No	5175 (94.87%)	2647 (95.01%)	1309 (95.48%)	1219 (96.21%)	
Yes	249 (4.56%)	139 (4.99%)	62 (4.52%)	48 (3.79%)	
GID					0.978
No	4464 (81.83%)	2290 (82.05%)	1129 (81.99%)	1045 (82.28%)	
Yes	974 (17.86%)	501 (17.95%)	248 (18.01%)	225 (17.72%)	

FBG, fasting blood glucose; Sbp, systolic blood pressure; Dbp, diastolic blood pressure; LDH, lactate dehydrogenase; RBP, Retinol-binding protein; TCH, total cholesterol; TG, triglyceride; TP, total protein; CRP, C-reactive protein; DBIL, direct bilirubin; TBIL, total bilirubin; ASHO, anti-streptolytic haemolysin O; HDL-C, High-density lipoprotein cholesterol; LDL-C, low-density lipoprotein cholesterol; APOB, Apolipoprotein B; ALT, alanine transaminase; AST, aspartate transaminase; ALP, alkaline phosphatase; CHE cholinesterase; PA, prealbumin; Cr, creatinine; UA, uric acid; GLO, globulin; MONO#, absolute monocyte count; BASO#, absolute basophil count; NEUT%, percentage of neutrophil; LYMH%, percentage of lymphocytes; MONO%, percentage of monocyte; EOS%, percentage of eosinophil; MPV, mean platelet volume; WBC, white blood cell; RBC, red blood cell; PLT, platelet; EOS#, absolute eosinophil count; BASO%, percentage of basophil; NEUT#, absolute neutrophil count; LYMH#, absolute lymphocytes count; HGB, haemoglobin; P-LCR, platelet-large cell ratio; BMI, body mass index; TyG, triglyceride-glucose; SII, systemic immune inflammation; FL, fractured limb; CRD, chronic respiratory disease; LD, liver disease; HD, heart disease; KD, kidney disease; GID, gastrointestinal disease.

**Figure 1:**
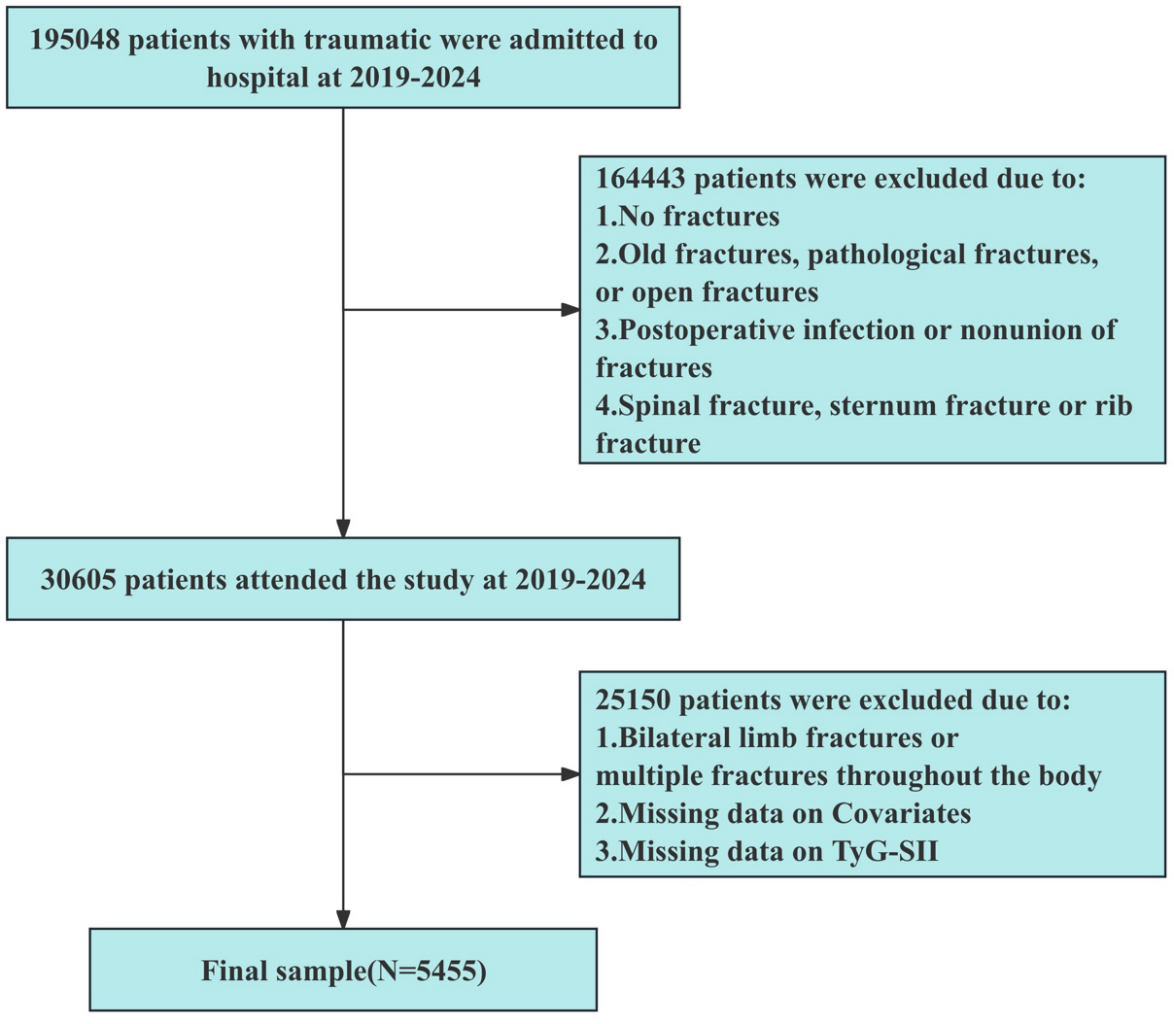
Flow chart of the study population.

Among these, 25150 individuals were excluded based on the following criteria: (1) simultaneous fractures in both upper and lower limbs or multiple fractures throughout the body; (2) incomplete covariate data (e.g., age, gender, medical history, vascular ultrasound results, laboratory biomarker results); (2) missing TyG index or SII data; (3) non-limb deep vein thrombosis. Ultimately, a total of 5,455 patients were included in the analysis (**Figure 1**).

### Exposure and outcome

Calculate the TyG index, SII index, and TyG-SII index using the following formulas: (1) TyG = ln [triglycerides (TG) (mg/dL) × fasting plasma glucose (FBG) (mg/dL)/2], (2) SII = platelet (PLT) (×10^9^/L) × neutrophil (NEUT#) (×10^9^/L)/lymph (LYMH#) (×10^9^/L), (3) TyG-SII = TyG × SII. A TyG-SII value less than 0 is classified as abnormal data and excluded from the study.

DVT is diagnosed using colour Doppler ultrasound as the preferred method, according to the specific guidelines (third edition) proposed by the Vascular Surgery Working Group of the Surgical Branch of the Chinese Medical Association in April 2017 (9). The primary endpoint of this study was preoperative venous thrombosis of the lower limbs. Based on duplex ultrasound findings, thrombotic events were categorized into two types: DVT involving the femoral vein and more distal deep veins, and muscular calf vein thrombosis (MCVT). Given the clinical relevance of MCVT in trauma patients—including its potential for proximal extension and risk of embolisation—MCVT was included in the composite primary endpoint alongside distal DVT. To examine potential heterogeneity in associations, the relationship between TyG-SII and distal DVT alone and MCVT alone was also evaluated separately in secondary analyses.

### Thrombosis prevention

In our institution, all patients presenting with traumatic fractures of the limbs or spine undergo colour Doppler ultrasound of the limb vessels within three days before surgery to screen for thrombus formation. The examination is typically scheduled to yield results by the third day of admission. Additionally, prophylactic measures are routinely initiated upon admission for all fracture patients, including subcutaneous administration of low-molecular-weight heparin (enoxaparin sodium), elevation of the affected limb, and active muscle contraction exercises.

If DVT is detected in the affected limb via Doppler ultrasound, interdisciplinary consultation is sought with the Departments of Radiology & Interventional Medicine and General Surgery. Management may involve intensifying low-molecular-weight heparin therapy or switching to oral rivaroxaban. A follow-up Doppler ultrasound is performed one week later to evaluate thrombus resolution. Provided that vascular recanalisation is confirmed, fracture surgery proceeds. If the thrombus persists, an inferior vena cava filter is usually placed in the femoral vein by the Interventional Radiology team prior to surgery. In select cases, patients may choose to accept the risk of thrombus embolism and request expedited surgical scheduling.

### Covariates

Based on the Chinese Guidelines for the Diagnosis and Treatment of Deep Vein Thrombosis (9), clinical experience, and evidence from previous literature on DVT risk factors, the corresponding covariates in the study data were selected. The variables included in the adjustment were gender, age, bedrest duration, alcohol consumption history, BMI, hypertension, hyperlipidaemia, diabetes, stroke, heart disease, renal disease, hepatic disease, rheumatoid arthritis, digestive system disease, chronic respiratory system disease, C-reactive protein, D-dimer, and antithrombin III, among others.

### Statistical analysis

**Figure 2:**
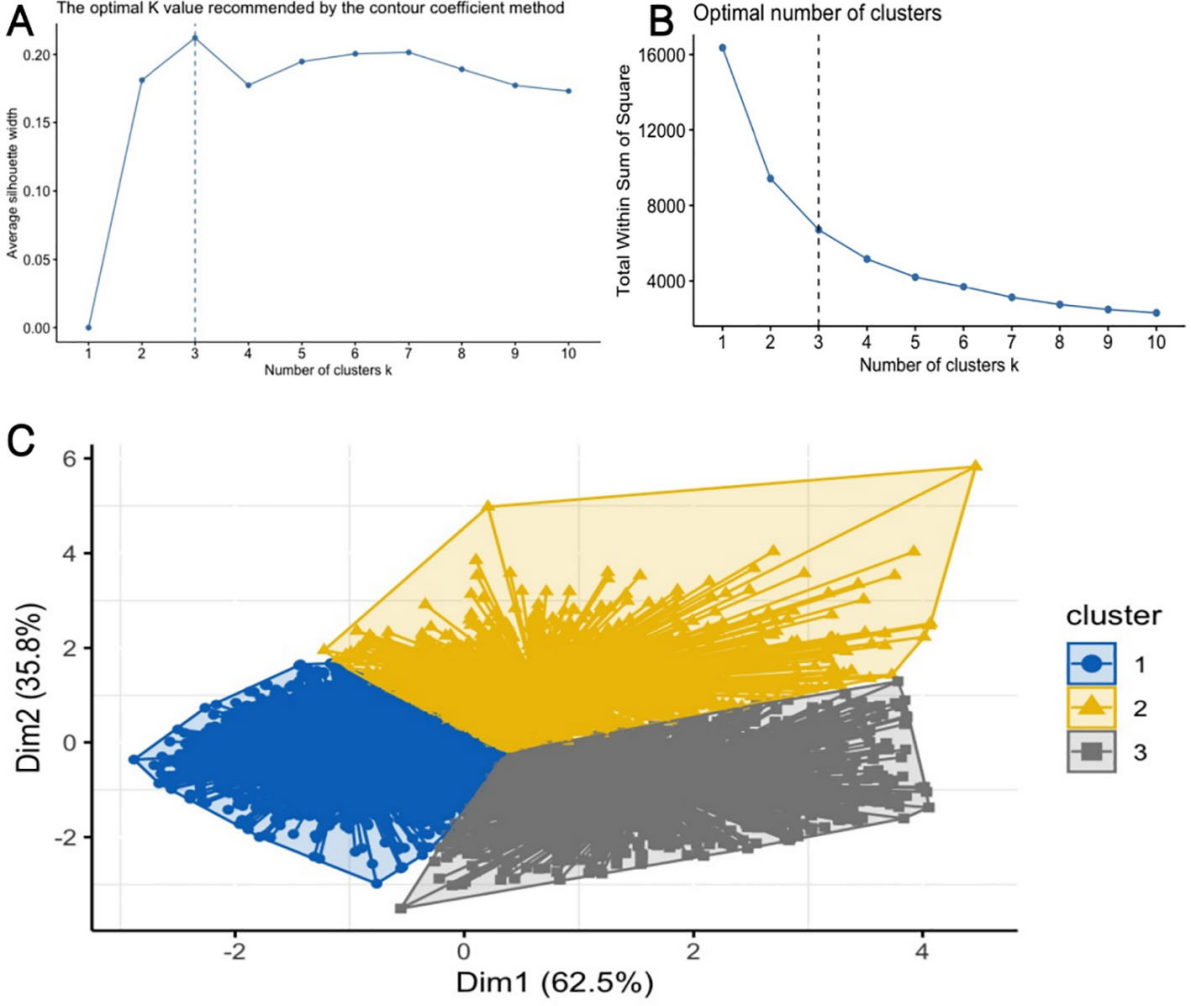
K-means clustering results. **(A, B)** Optimal classification K value, **(C)** Clustering diagrams for TyG-SII.

We used K-means clustering to categorize patients into three groups based on TyG-SII dynamics (low, moderate, high). Additionally, to evaluate the dose-response relationship between baseline TyG-SII and DVT risk, patients were stratified into quartiles (Q1–Q4) of baseline TyG-SII. K-means clustering is a widely used unsupervised learning algorithm, implemented using the cluster and factor extra packages. We used the optimal K value and the elbow method to determine the appropriate number of classifications for TyG-SII changes (**Figure 2**). Based on the baseline TyG-SII change levels, patients were divided into three groups: the first group (n=2795) represented low-level TyG-SII; the second group (n=1383) represented moderate-level TyG-SII; and the third group (n=1276) represented high-level TyG-SII. Continuous variables were expressed as mean (standard deviation, SD) or median (interquartile range, IQR), while categorical variables were expressed as frequency (proportion). We used multivariate binary logistic regression analysis to examine the association between different clusters and DVT and MCVT. Results were presented as odds ratios (ORs) and 95% confidence intervals (CIs). Three models were estimated: Model 1 was unadjusted for any covariates. Model 2 adjusted for gender, age, alcohol history, BMI, hypertension, hyperlipidaemia, and diabetes. Model 3 further adjusted for stroke, heart disease, renal disease, hepatic disease, rheumatoid arthritis, gastrointestinal disease, chronic respiratory disease, D-dimer, and antithrombin III. Additionally, patients were divided into four groups based on the quartiles of baseline TyG-SII to examine the relationship between TyG-SII and DVT and MCVT. The trend P-values were calculated by determining the median TyG-SII within each quartile. Furthermore, we conducted subgroup analyses and interaction analyses to investigate whether the relationship between TyG-SII and DVT/MCVT varied across specific populations (e.g., age, gender, BMI, alcohol consumption, etc.). The above results were achieved using the scitable and survival packages. We also used restricted cubic spline (RCS) regression models to explore potential nonlinear associations between TyG-SII and DVT and MCVT events, and further calculated thresholds for nonlinear relationships. Receiver operating characteristic (ROC) curves were used for diagnostic value analysis, and the area under the curve was calculated to quantify the predictive ability of TyG, SII, TyG-SII for DVT, and TyG-SII for MCVT. A two-sided P value < 0.05 was considered statistically significant. All analyses were performed on R software-4.3.2 (2023.12.1 + 402, R Development Core team).

## Results

### Baseline data

In our study, a total of 5,455 patients were included for analysis. The mean age at baseline was 48.08 (± 15.55) years, the mean height was 158.40 (± 8.75) cm, the mean weight was 58.26 (± 11.46) kg, and the mean BMI was 23.16 (± 3.95) kg/m². Among them, 3,112 (57.05%) were male, 2,343 (42.95%) were female, 3,073 (56.33%) patients had fractures in the left limb, 2,382 (43.67%) patients had fractures in the right limb, and 3,252 (59.62%) patients consumed alcohol. The average blood pressure was 130.50 (± 22.07)/76.06 (± 12.52) mmHg, the average FBG was 6.00 (± 1.90) mmol/L, and the average TyG was 1.22 (± 0.60). the average SII was 876.75 (± 619.45), the average TCH was 4.70 (± 1.12) mmol/L, the average TG were 1.35 (± 0.95) mmol/L, the average TP was 68.26 (± 6.11) g/L, mean CRP was 30.45 (± 33.04) mg/L, mean HDL-C was 1.29 (± 0.24) mmol/L, mean LDL-C was 68.26 (± 6.11) mmol/L, mean ALT was 30.02 (± 34.80) U/L, average AST was 29.11 (± 28.52) U/L, average ALP was 78.98 (± 32.63) U/L, the average Cr was 70.88 (± 33.03) μmol/L, the average UA was 322.73 (± 104.23) μmol/L, the average Na level was 140.61 (± 2.67) mmol/L, the average Cl level was 102.78 (± 2.90) mmol/L, the average WBC count was 8.59 (± 2.67) ×10^9/L, the average RBC count was 4.27 (± 0.79) ×10^12/L, the average PLT was 245.18 (± 78.55) ×10^9/L, the average NEUT# was 5.90 (± 2.37) ×10^9/L, the average LYMH# was 1.83 (± 0.65) ×10^9/L, the average HGB was 121.47 (± 21.10) g/L, and the average D-dimer was 5.99 (± 9.26) μg/ml. Additionally, 1,290 (23.65%) had hypertension, 378 (6.93%) had hyperlipidaemia, 252 (4.62%) had diabetes, 35 (0.64%) had cancer, 504 (9.24%) had chronic respiratory diseases, 188 (3.45%) had Asthma, 214 (3.92%) had liver disease, 90 (1.65%) had stroke, 507 (9.29%) had heart disease, 249 (4.56%) had kidney disease, and 974 (17.86%) had gastrointestinal disease. Based on cluster analysis, additional baseline characteristics of each group are presented in **Table 1**.

### DVT and MCVT risk prediction

**Figure 3:**
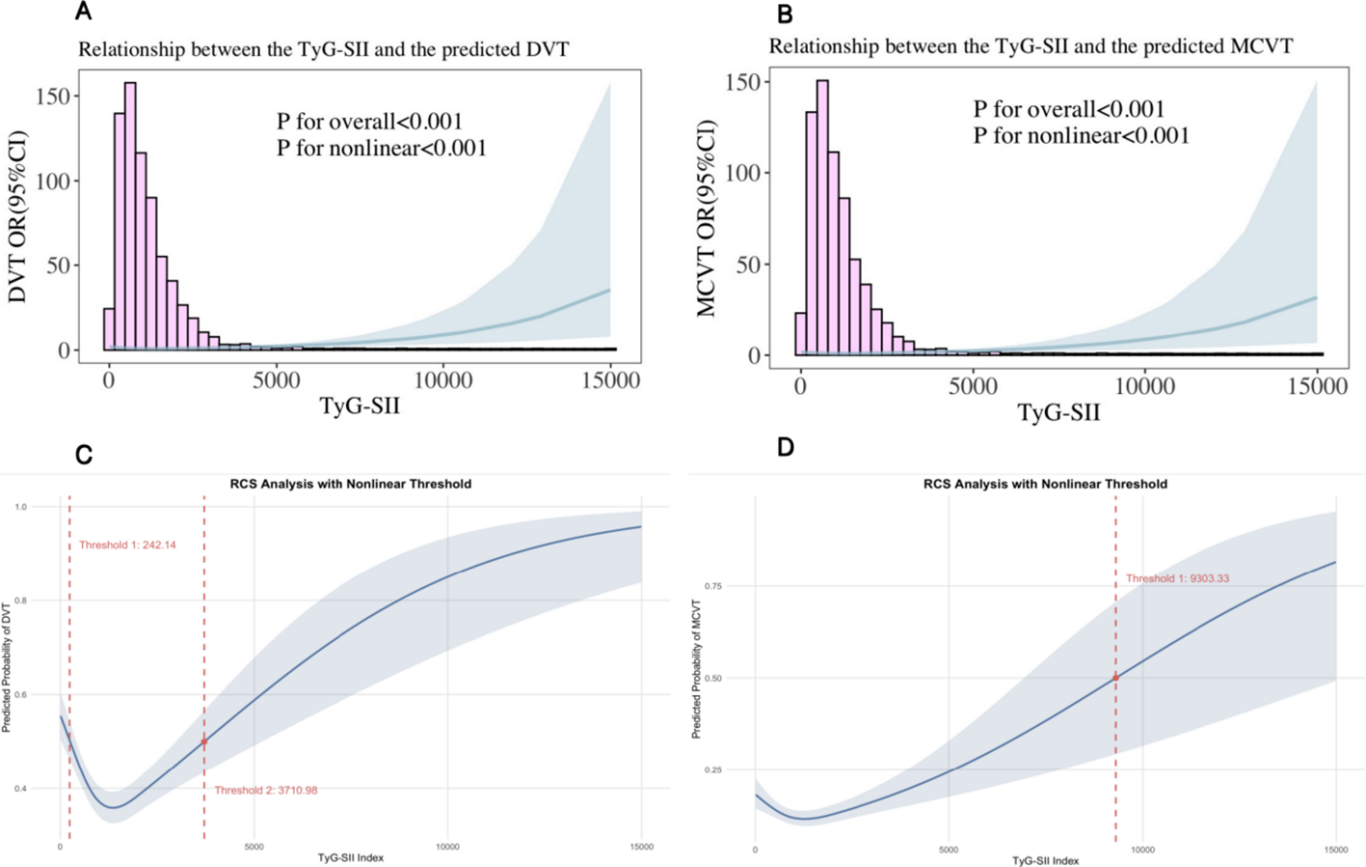
RCS plots depicting the nonlinear association between baseline TyG-SII and the risk of **(A)** DVT and **(B)** MCVT, the nonlinear threshold between baseline TyG-SII and the risk of **(C)** DVT, and **(D)** MCVT, based on fully adjusted Model 3. The RCS models were fitted with 4 knots placed at the 5th, 35th, 65th, and 95th percentiles of TyG-SII distribution. The shaded areas represent 95% confidence intervals.

During the period from hospital admission to surgery, 1,991 (36.50%) patients developed DVT. The results in **Table 2** present the multi-model logistic regression analysis results of the association between baseline TyG-SII and its dynamic changes and DVT risk. In Model 1, which did not adjust for confounding factors, compared with the low-level category (Cluster 1), individuals with moderate-level (Cluster 2) and high-level (Cluster 3) TyG-SII had a 7% lower risk of DVT (OR = 0.93, 95% CI: 0.81–1.06, P = 0.2807) and 22% (OR = 0.78, 95% CI: 0.69–0.90, P = 0.0006), respectively, compared with the low-level category (Cluster 1). After stepwise adjustment for confounding factors, Model 3 showed that the protective effects of Cluster 2 and Cluster 3 remained significant, with risk reductions of 14% (OR = 0.86, 95% CI: 0.66–0.98, P = 0.0434) and 27% (OR = 0.73, 95% CI: 0.58–0.95, P = 0.0001). Further analysis of baseline TyG-SII revealed a nonlinear association: in Model 3, which fully adjusted for confounding factors, the third quartile (Q3) showed the most significant protective effect, with a 54% reduction in DVT risk compared to the lowest quartile (Q1) (OR = 0.46, 95% CI: 0.39–0.55, P < 0.0001), while the highest quartile (Q4) had a 17% higher risk compared to the third quartile (Q3) (OR = 0.63, 95% CI: 0.53–0.76, P < 0.0001). Notably, the risk associated with each standard deviation increase in TyG-SII was not statistically significant (P = 0.2787), indicating that the impact of metabolic indicators on DVT risk exhibits trajectory dependence and threshold effects. The RCS regression model (**Figures 3A, C**) showed a nonlinear U-shaped relationship between baseline TyG-SII and DVT risk in the fully adjusted Model 3 (P for nonlinear <0.001), with two thresholds at 242.14 and 3710.98.

**Table 2:** Different cluster of TyG-SII, baseline TyG-SII and risk of DVT.

Characters	Statistics	Model.1		Model.2		Model.3	
		OR(95%CI)	P value	OR(95%CI)	P value	OR(95%CI)	P value
Change in the TyG-SII							
Cluster 1	2796 (51.26%)	1.0(Ref)		1.0(Ref)		1.0(Ref)	
Cluster 2	1383 (25.35%)	0.93 (0.81, 1.06)	0.2807	0.86 (0.74, 1.00)	0.0483	0.86 (0.73, 1.00)	0.0434
Cluster 3	1276 (23.39%)	0.78 (0.68, 0.90)	0.0006	0.74 (0.64, 0.86)	0.0001	0.73 (0.62, 0.85)	<0.0001
Baseline TyG-SII							
Q1	1364 (25.00%)	1.0(Ref)		1.0(Ref)		1.0(Ref)	
Q2	1361 (24.95%)	0.76 (0.65, 0.88)	0.0004	0.74 (0.63, 0.88)	0.0006	0.73 (0.62, 0.87)	0.0005
Q3	1362 (24.97%)	0.50 (0.43, 0.59)	<0.0001	0.48 (0.40, 0.57)	<0.0001	0.46 (0.39, 0.55)	<0.0001
Q4	1368 (25.08%)	0.87 (0.74, 1.01)	0.0640	0.72 (0.60, 0.86)	0.0002	0.63 (0.53, 0.76)	<0.0001
P for trend			0.0010		<0.0001		<0.0001
Per SD increase		1.04 (0.99, 1.10)	0.1563	1.00 (0.94, 1.07)	0.9177	0.96 (0.90, 1.03)	0.2787

During the period from hospital admission to surgery, 646 (11.84%) patients developed MCVT. The results in **Table 3** present the findings of a multi-model logistic regression analysis examining the association between baseline TyG-BMI and its dynamic changes with MCVT risk. Compared with Model 1 (unadjusted for confounders) and Model 3 (fully adjusted for confounders), Model 2 (partially adjusted for confounders) showed the lowest MCVT risk in individuals with moderate (Cluster 2) and high (Cluster 3) TyG-SII levels (OR = 0.76). Further analysis of baseline TyG-SII revealed a nonlinear association: in both Model 2 (partially adjusted for confounders) and Model 3 (fully adjusted for confounders), the third quartile (Q3) showed the most significant protective effect, with a 44% reduction in MCVT risk compared to the lowest quartile (Q1) (OR = 0.56). Additionally, the highest quartile (Q4) showed an increase compared to the third quartile (Q3), but the risk increase per standard deviation of TyG-SII was not statistically significant (all P > 0.05), indicating that the impact of metabolic indicators on MCVT risk exhibits trajectory dependence and threshold effects. The RCS regression model (**Figures 3B, D**) showed a nonlinear U-shaped relationship between baseline TyG-SII and MCVT risk in the fully adjusted Model 3 (P for nonlinear <0.001), with a threshold of 9303.33.

**Table 3:** Different cluster of TyG-SII, baseline TyG-SII and risk of MCVT.

Characters	Statistics	Model.1		Model.2		Model.3	
		OR(95%CI)	P value	OR(95%CI)	P value	OR(95%CI)	P value
Change in the TyG-SII							
Cluster 1	2796 (51.26%)	1.0(Ref)		1.0(Ref)		1.0(Ref)	
Cluster 2	1383 (25.35%)	0.83 (0.68, 1.01)	0.0647	0.76 (0.62, 0.94)	0.0112	0.78 (0.63, 0.96)	0.0219
Cluster 3	1276 (23.39%)	0.79 (0.64, 0.98)	0.0293	0.76 (0.61, 0.94)	0.0107	0.77 (0.62, 0.97)	0.0236
Baseline TyG-SII							
Q1	1364 (25.00%)	1.0(Ref)		1.0(Ref)		1.0(Ref)	
Q2	1361 (24.95%)	1.03 (0.83, 1.29)	0.7613	1.00 (0.80, 1.26)	0.9799	1.00 (0.79, 1.27)	0.9818
Q3	1362 (24.97%)	0.58 (0.45, 0.74)	<0.0001	0.56 (0.44, 0.73)	<0.0001	0.56 (0.43, 0.73)	<0.0001
Q4	1368 (25.08%)	0.94 (0.75, 1.18)	0.5865	0.80 (0.63, 1.01)	0.0617	0.86 (0.67, 1.10)	0.2254
P for trend			0.0526		0.0018		0.0107
Per SD increase		1.07 (0.99, 1.15)	0.0993	1.03 (0.94, 1.12)	0.5302	1.07 (0.98, 1.16)	0.1375

**Figure 4:**
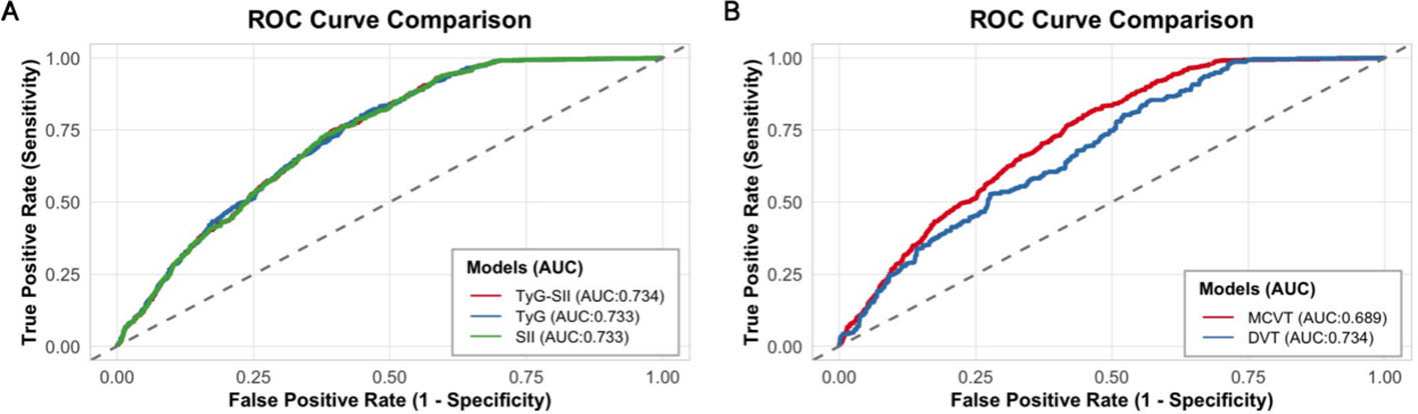
Receiver operating characteristic curves for **(A)** baseline TyG-SII, TyG, and SII predicting DVT and **(B)** baseline TyG-SII predicting DVT and MCVT.

ROC curves (**Figure 4**) showed that TyG-SII (AUC 0.734, 95% CI: 0.721–0.749, P < 0.001) has the highest diagnostic performance for DVT, followed by TyG (AUC 0.733, 95% CI: 0.719–0.747, P < 0.001) and SII (AUC 0.733, 95% CI: 0.719–0.747, P < 0.001), while TyG-SII had lower diagnostic efficacy for MCVT risk (AUC 0.689, 95% CI: 0.668–0.710, P < 0.001) compared to DVT risk.

### Subgroup analysis

**Figure 5:**
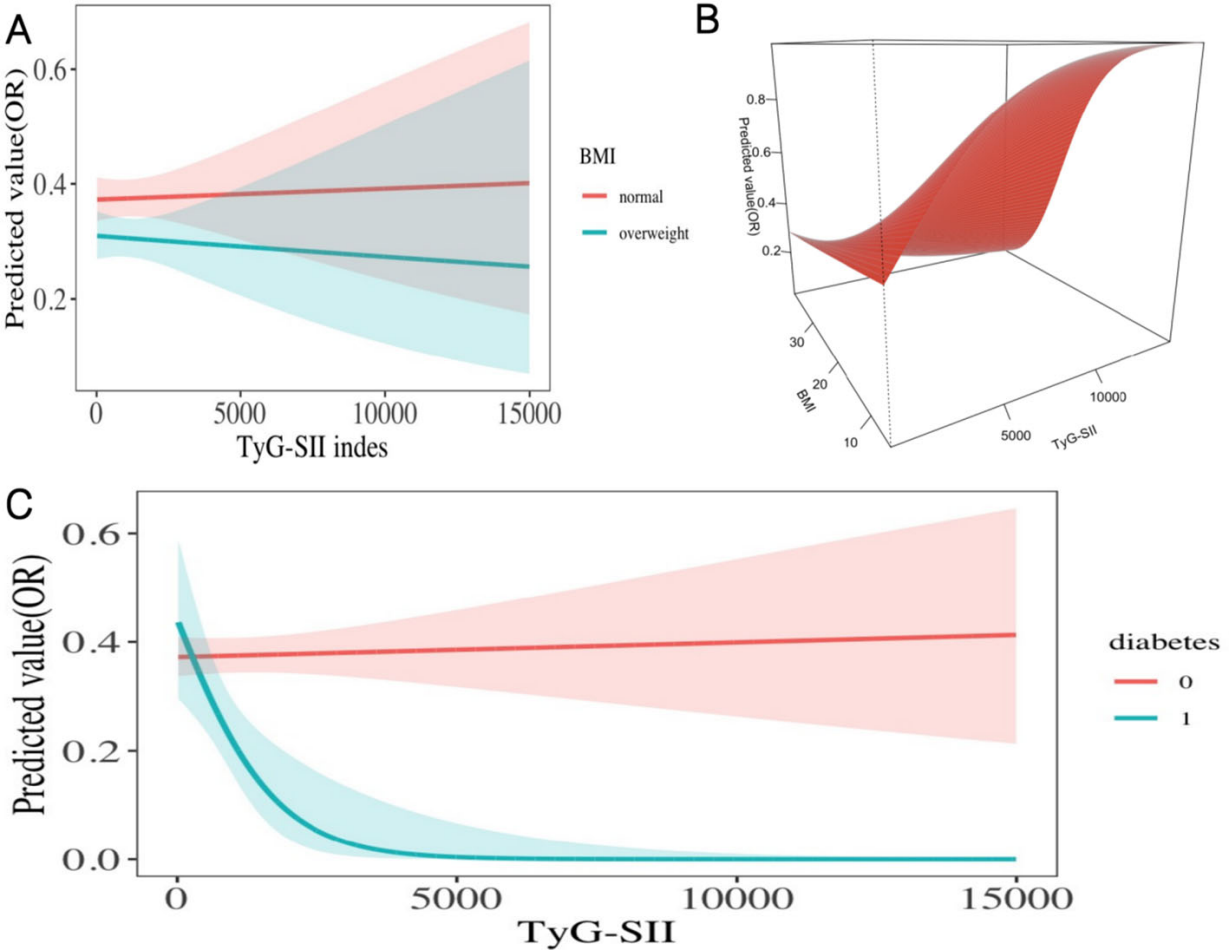
Interaction effect model of the association between baseline TyG-SII predicted risk for DVT and **(A, B)** BMI; **(C)** diabetes.

Subgroup analysis of DVT risk (**Table 4**, **Figure 5**) showed that, after fully adjusting for confounding factors, there was a significant interaction between TyG-SII dynamic levels and BMI (P for interaction = 0.013), and the interaction between baseline TyG-SII and BMI was also significant (P for interaction = 0.031). In the normal BMI group (<24 kg/m²), Cluster 3 (high-level group) exhibited the strongest protective effect (OR = 0.72, 95% CI: 0.60–0.88), while baseline TyG-SII showed a U-shaped association: Q3 showed a protective peak (OR = 0.55, 95% CI: 0.44–0.69), but the effect weakened in Q4 (OR = 0.84, 95% CI: 0.67–1.06). For the high-risk group (≥24 kg/m²), Cluster 2 (moderate-level group) showed the most significant protective effect (OR = 0.63, 95% CI: 0.48–0.83), and baseline TyG-SII demonstrated dose-dependent protection: Q3 and Q4 both achieved equivalent strong protective levels (OR = 0.47), and the risk continued to decrease with increasing TyG-SII (P for trend < 0.001), forming a monotonically decreasing curve (**Figure 5B**), with protection enhanced by 37% (ΔOR = 0.37) compared to the normal weight group at the Q4 level. Notably, diabetic patients exhibited a more pronounced dose-response relationship: at the Q4 level, the DVT risk in the diabetic group was reduced by 91% (OR = 0.09, 95% CI: 0.02–0.38), significantly superior to the non-diabetic group (OR = 0.72, 95% CI: 0.60–0.87; P for interaction = 0.007). This enhanced protective effect was visually validated in the interaction plot (**Figure 5C**), where the OR curve for the diabetic group sharply declined when TyG-SII > 10,000. Additionally, patients with hyperlipidaemia exhibited an unconventional response (Q2 OR = 2.05), suggesting that severe metabolic disorders may reverse the protective effect of TyG-SII. No significant interactions were observed in other subgroups, indicating that these factors may not substantially alter the association between TyG-SII and DVT.

**Table 4:** Subgroup analysis for the association (OR, 95% CI) between TyG-SII and risk for DVT.

Subgroup	Change in the TyG-SII				Baseline TyG-SII					
	Cluster 1	Cluster 2	Cluster 3	P for interaction	Q1	Q2	Q3	Q4	P for trend	P for interaction
Age				0.922						0.792
< 45 years	Ref	0.87 (0.68, 1.12)	0.75 (0.58, 0.96)		Ref	0.80 (0.61, 1.06)	0.53 (0.40, 0.71)	0.61 (0.45, 0.83)	<0.001	
≥ 45 years	Ref	0.82 (0.67, 0.99)	0.72 (0.59, 0.88)		Ref	0.79 (0.63, 1.00)	0.49 (0.38, 0.62)	0.69 (0.54, 0.87)	0.003	
Sex				0.592						0.197
Female	Ref	0.91 (0.71, 1.16)	0.70 (0.55, 0.90)		Ref	0.82 (0.63, 1.07)	0.44 (0.33, 0.58)	0.67 (0.49, 0.90)	0.001	
Male	Ref	0.82 (0.67, 1.00)	0.77 (0.62, 0.94)		Ref	0.83 (0.66, 1.05)	0.63 (0.49, 0.80)	0.72 (0.57, 0.92)	0.008	
BMI				0.013						0.031
< 24 kg/m²	Ref	0.99 (0.82, 1.19)	0.72 (0.60, 0.88)		Ref	0.86 (0.70, 1.07)	0.55 (0.44, 0.69)	0.84 (0.67, 1.06)	0.114	
≥ 24 kg/m²	Ref	0.63 (0.48, 0.83)	0.77 (0.59, 1.01)		Ref	0.73 (0.54, 1.00)	0.47 (0.34, 0.64)	0.47 (0.34, 0.65)	<0.001	
FL				0.416						0.116
right	Ref	0.80 (0.63, 1.02)	0.66 (0.51, 0.84)		Ref	0.64 (0.49, 0.84)	0.42 (0.32, 0.56)	0.57 (0.43, 0.76)	<0.001	
left	Ref	0.89 (0.73, 1.08)	0.81 (0.66, 1.00)		Ref	0.94 (0.75, 1.19)	0.61 (0.48, 0.78)	0.78 (0.62, 0.99)	0.009	
Hypertension				0.998						0.297
No	Ref	0.84 (0.71, 1.00)	0.74 (0.62, 0.89)		Ref	0.82 (0.67, 1.00)	0.51 (0.42, 0.63)	0.75 (0.61, 0.93)	0.004	
Yes	Ref	0.83 (0.60, 1.16)	0.74 (0.53, 1.02)		Ref	0.81 (0.56, 1.18)	0.61 (0.41, 0.91)	0.58 (0.39, 0.86)	0.005	
hyperlipidaemia				0.535						0.063
No	Ref	0.87 (0.74, 1.01)	0.73 (0.62, 0.86)		Ref	0.78 (0.65, 0.93)	0.53 (0.44, 0.64)	0.71 (0.59, 0.86)	<0.001	
Yes	Ref	0.70 (0.32, 1.51)	0.99 (0.47, 2.08)		Ref	2.05 (0.92, 4.57)	0.70 (0.27, 1.77)	0.63 (0.21, 1.90)	0.130	
Diabetes				0.104						0.007
No	Ref	0.86 (0.74, 1.01)	0.75 (0.64, 0.88)		Ref	0.84 (0.70, 1.00)	0.55 (0.46, 0.66)	0.72 (0.60, 0.87)	<0.001	
Yes	Ref	0.28 (0.09, 0.85)	0.38 (0.14, 1.05)		Ref	0.93 (0.29, 3.00)	0.15 (0.04, 0.54)	0.09 (0.02, 0.38)	<0.001	

**Figure 6:**
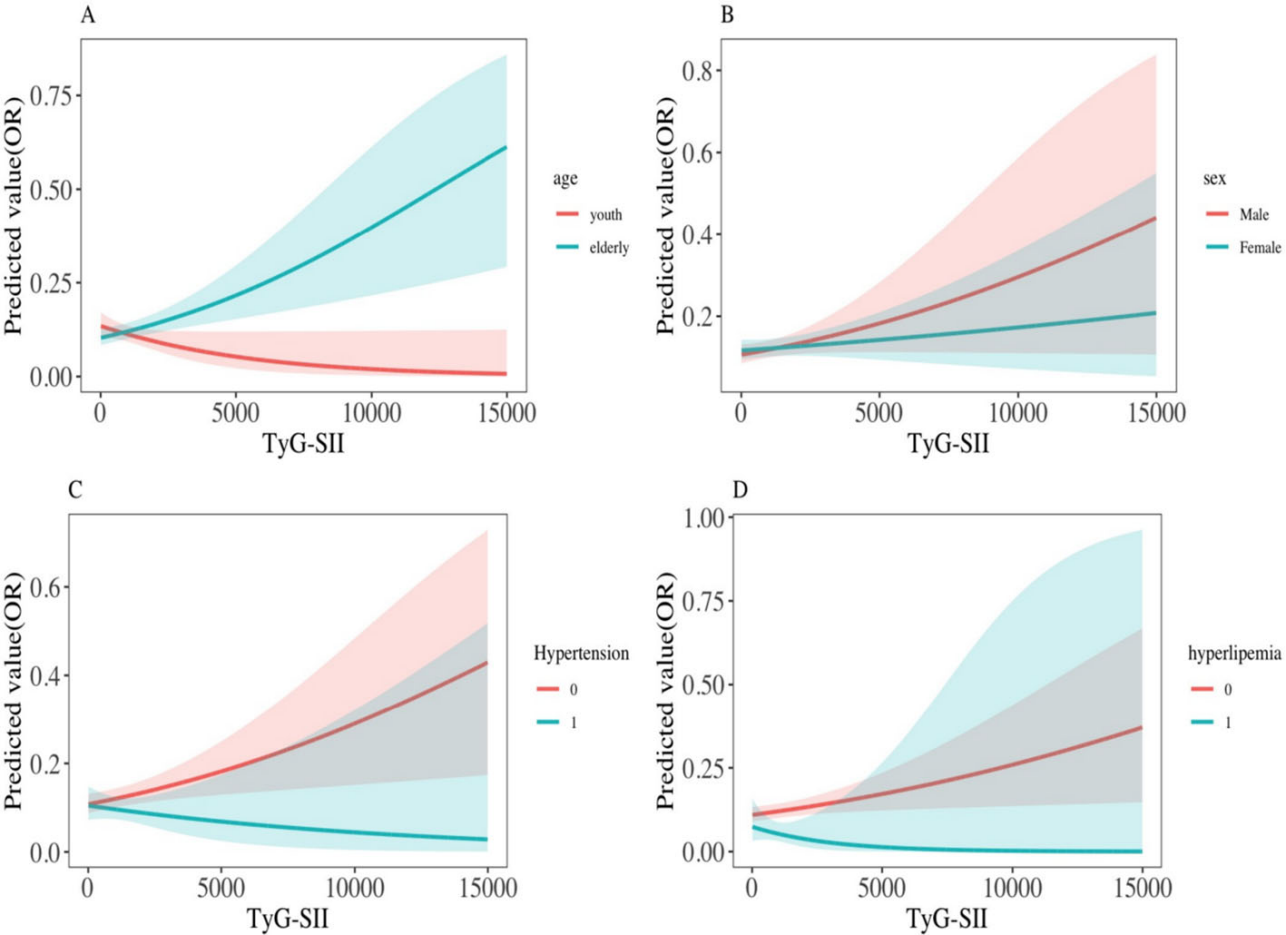
Interaction effect model of the association between baseline TyG-SII predicted risk for DVT and **(A)** age, **(B)** sex, **(C)** hypertension, **(D)** hyperlipidaemia.

Subgroup analysis of MCVT risk (**Table 5**, **Figure 6**) revealed that, after fully adjusting for confounding factors, the association between different levels of TyG-SII (Cluster 1, 2, 3) and MCVT risk was significantly modulated by diabetes status (P for interaction = 0.001). In diabetic patients, Cluster 2 (moderate level) exhibited a strong protective effect (OR = 0.25, 95% CI: 0.02–3.57), but the interaction with baseline TyG-SII was not significant (P for interaction = 0.164). Baseline TyG-SII showed significant interactions with age, sex, hypertension, and hyperlipidaemia (all P for interaction <0.05). Age stratification revealed important differences: the strongest protective effect was observed in the young group aged <45 years (OR = 0.51, 95% CI: 0.31–0.82; P for trend <0.05), while no such trend was observed in the middle-aged and elderly group aged ≥45 years (P for trend =0.530). **Figure 6A** visually demonstrates that the OR curves for the young group and the middle-aged and elderly group exhibit completely opposite trends. Gender-stratified effects were also significant: females in Cluster 2 (OR = 0.64, 95% CI: 0.45–0.91) and Q3 (OR = 0.36, 95% CI: 0.24–0.57), while men showed no significant association (Q3 OR = 0.89, 95% CI: 0.62–1.26). **Figure 6B** visually shows that the OR curve for women remains consistently lower than that for men. Hypertensive status significantly modified the baseline TyG-SII effect (P for interaction <0.001): the non-hypertensive group showed significant protection in Q3 (OR = 0.53, 95% CI: 0.39–0.71), while the hypertensive group exhibited an abnormal increase in risk in Q2 (OR = 2.00, 95% CI: 1.11–3.59), followed by decreases in Q3, Q4 showed a decrease compared to previous levels, which is similar to the overall trend observed in the hypertension group in **Figure 6C** of the dose-response curve—the risk sharply decreases when TyG-SII > 5000. However, the reliability of data for hyperlipidaemia patients was limited (OR values were infinite), but the non-patient group showed significant protection in Q3 (OR = 0.56, 95% CI: 0.43–0.73). However, changes indicated in **Figure 6D** suggest that the risk of MCVT is lower in the hyperlipidaemia patient group.

**Table 5:** Subgroup analysis for the association (OR, 95% CI) between TyG-SII and risk for MCVT.

Subgroup	Change in the TyG-SII				Baseline TyG-SII					
	Cluster 1	Cluster 2	Cluster 3	P for Inter- action	Q1	Q2	Q3	Q4	P for trend	P for Inter- action
Age				0.330						<0.001
< 45 years	Ref	0.62 (0.42, 0.91)	0.82 (0.57, 1.19)		Ref	1.34 (0.92, 1.94)	0.66 (0.43, 1.01)	0.51 (0.31, 0.82)	<0.001	
≥ 45 years	Ref	0.84 (0.64, 1.10)	0.75 (0.56, 0.99)		Ref	0.94 (0.68, 1.28)	0.50 (0.35, 0.72)	1.08 (0.79, 1.47)	0.530	
Sex				0.333						0.017
Female	Ref	0.64 (0.45, 0.91)	0.69 (0.48, 0.97)		Ref	0.87 (0.61, 1.25)	0.36 (0.24, 0.57)	0.82 (0.55, 1.21)	0.147	
Male	Ref	0.86 (0.65, 1.15)	0.88 (0.65, 1.18)		Ref	1.35 (0.97, 1.87)	0.89 (0.62, 1.26)	1.11 (0.79, 1.56)	0.863	
BMI				0.247						0.664
< 24 kg/m²	Ref	0.89 (0.68, 1.16)	0.81 (0.61, 1.07)		Ref	1.11 (0.82, 1.50)	0.65 (0.46, 0.90)	1.03 (0.75, 1.40)	0.739	
≥ 24 kg/m²	Ref	0.59 (0.39, 0.89)	0.69 (0.47, 1.02)		Ref	1.26 (0.83, 1.92)	0.56 (0.35, 0.90)	0.85 (0.54, 1.35)	0.146	
FL				0.089						0.954
right	Ref	0.62 (0.43, 0.88)	0.60 (0.42, 0.87)		Ref	1.15 (0.80, 1.66)	0.62 (0.41, 0.94)	0.90 (0.60, 1.35)	0.219	
left	Ref	0.88 (0.66, 1.16)	0.96 (0.72, 1.27)		Ref	1.09 (0.79, 1.51)	0.59 (0.41, 0.85)	0.97 (0.70, 1.35)	0.528	
Hypertension				0.122						<0.001
No	Ref	0.70 (0.54, 0.89)	0.82 (0.64, 1.05)		Ref	0.93 (0.71, 1.22)	0.53 (0.39, 0.71)	1.02 (0.78, 1.34)	0.923	
Yes	Ref	0.99 (0.61, 1.62)	0.58 (0.34, 1.00)		Ref	2.00 (1.11, 3.59)	0.97 (0.50, 1.87)	0.65 (0.33, 1.28)	0.018	
Hyperlipidaemia				0.740						0.002
No	Ref	0.76 (0.61, 0.95)	0.77 (0.61, 0.97)		Ref	1.04 (0.81, 1.32)	0.56 (0.43, 0.73)	0.92 (0.71, 1.18)	0.195	
Yes	Ref	0.65 (0.14, 3.00)	1.29 (0.30, 5.63)		Ref	Inf (0.00, Inf)	Inf (0.00, Inf)	Inf (0.00, Inf)	0.532	
Diabetes				0.001						0.164
No	Ref	0.76 (0.61, 0.94)	0.80 (0.64, 1.00)		Ref	1.08 (0.85, 1.37)	0.58 (0.44, 0.76)	0.93 (0.72, 1.19)	0.188	
Yes	Ref	0.25 (0.02, 3.57)	0.00 (0.00, Inf)		Ref	2.83 (0.09, 85.13)	1.73 (0.06, 50.40)	0.16 (0.00, 6.30)	0.150	

## Discussions

In this study, we first observed the relationship between the dynamic changes in TyG-SII and baseline TyG-SII and the risk of DVT/MCVT in a population with traumatic fractures. The results showed that both high levels of TyG-SII and higher baseline TyG-SII were associated with a lower incidence of DVT/MCVT events, exhibiting a negative nonlinear relationship. Additionally, compared to baseline SII and TyG, TyG-SII demonstrated higher accuracy in predicting DVT risk and was superior in predicting MCVT risk. Furthermore, TyG-SII was associated with lower DVT risk in overweight and diabetic populations and lower MCVT risk in hypertensive and hyperlipidaemic populations; however, overall MCVT incidence risk was higher in middle-aged and elderly female patients.

### Pathogenesis of DVT in patients with traumatic fractures

German physician Rudolf Carl Virchow (1821–1902) first proposed ‘Virchow’s Triad’ in 1856, referring to the three elements of thrombus formation: stasis, endothelial injury, and hypercoagulable state (20). Clinically, conditions such as hemiplegia after stroke, plaster cast immobilisation after fracture, and prolonged bed rest after spinal cord injury can lead to low shear stress in the venous valve sinuses, resulting in blood stasis and hypoxia. This activates vascular endothelial cells to release P-selectin and tissue factor (TF), promoting platelet adhesion (21). Vascular injury, caused by surgery, infection, or hypoxia, disrupts endothelial glycocalyx through oxidative stress, reduces anticoagulant substances (such as thrombomodulin and heparan sulphate), exposes subendothelial collagen, and initiates the coagulation cascade, thereby promoting the coagulation process (21, 23, 24). Finally, genetic anticoagulant protein defects (protein C/S deficiency) or acquired factors (such as malignant tumours) that enhance thrombin generation, platelet function, and abnormalities in the coagulation and fibrinolysis pathways all contribute to hypercoagulability (21, 25, 26). Currently, researchers generally agree that the presence of two or more risk factors significantly increases the risk of DVT, particularly when venous stasis is the core factor contributing to DVT.

Traumatic fracture patients, due to the effects of violent trauma, may experience indirect damage to soft tissues when the force is transmitted to the bones. The blood vessels located between the soft tissues may also sustain corresponding damage. Although closed fracture patients and fracture fragments do not directly damage blood vessels, noticeable subcutaneous bruising may still appear locally, which is an indication of capillary damage. Additionally, in some fracture patients, significant displacement of the fracture ends can cause direct contusion of deep vessels, such as in tibia-fibula double-stem fractures, femoral shaft fractures, and radius-ulna double-stem fractures, where displacement is particularly pronounced after loss of shaft support, making direct vascular damage more likely. Therefore, the primary risk factor for DVT in traumatic fracture patients is vascular endothelial damage. Secondly, after a fracture, bleeding from microvascular and capillary damage, as well as bone marrow cavity bleeding, can exert significant internal pressure on the fracture ends (manifesting as local swelling). This pressure continuously compresses blood vessels within soft tissues. On one hand, compressed vessels narrow, leading to blood stasis; on the other hand, for patients with pre-existing vascular damage or underlying vascular diseases, this further exacerbates the extent of vascular endothelial damage. Additionally, to limit the continued damage to soft tissues caused by the fracture ends, clinical practice commonly employs plaster cast immobilisation or bone traction to stabilise the injured limb. During this period, the injured limb remains in a state of prolonged immobilisation, lacking normal functional activity, which leads to blood stasis in the distal limb and increases the risk of DVT. The mechanisms and processes of local bleeding and coagulation following a fracture have been detailed in previous literature (22, 23). During this process, the blood circulation in the injured limb is in a hypercoagulable state, further increasing the risk of thrombus formation.

### Application of immune-inflammatory response index in DVT

The process of bleeding from a fracture to haemostasis described earlier is a physiological process triggered by pathological injury. Researchers have found that this process involves an intrinsic effect mechanism of the innate immune system, which is also present during thrombus formation, referred to as immune thrombosis (27). This intrinsic effect produces pro-inflammatory cytokines, which further stimulate the production of inflammatory biomarkers, forming the ‘immune-inflammatory-coagulation axis’ (28, 29). Breakthroughs in research after 2015 revealed the profound regulation of the immune-inflammatory response on the Virchow triangle: following endothelial injury, neutrophils release neutrophil extracellular traps (NETs), providing a fibrin deposition scaffold and activating the extrinsic coagulation pathway via tissue factor (21, 24); platelets form aggregates with neutrophils via the P-selectin-PSGL-1 axis, accelerating microthrombus growth, with their phosphatidylserine (PS) externalisation significantly enhancing prothrombinase complex efficiency (21); Monocytes secrete tissue factor (TF) and inflammatory factors (such as MCP-1), driving venous wall fibrosis (24). This synergistic interaction establishes a pre-thrombotic microenvironment through combined coagulation activation, inflammatory cytokine release, and vascular permeability regulation. This stage also reveals a coagulation-inflammation vicious cycle: thrombin activates protease activation receptors (PARs) to amplify inflammatory signals, while chronic inflammatory diseases increase the risk of DVT recurrence through sustained endothelial damage (24). Therefore, an increasing number of researchers are focusing on the application of immune-inflammatory response indices in assessing DVT risk in patients with traumatic fractures. A retrospective study of preoperative DVT in patients with intertrochanteric femoral fractures found that, through a multivariate logistic regression model, body mass index (BMI) (OR 0.79, 95% CI 0.63–0.99, P = 0.042), neutrophil-to-lymphocyte ratio (NLR) (OR 7.29, 95% CI 1.53, 34.64, P = 0.0012), and systemic immune inflammation index (SII) (OR 6.61, 95% CI 2.35–18.59, P = 0.001) were independent predictors of preoperative DVT in patients with intertrochanteric femoral fractures (30). Another study on total joint arthroplasty (TJA) patients found that not only were higher SII and NLR independent risk factors for preoperative DVT in TJA patients, but high levels of MLR and PLR were also high-risk factors for DVT (11). Additionally, a study on postoperative DVT in total knee arthroplasty (TKA) patients found that the aforementioned inflammatory response indices (SII, NLR, MLR, and PLR) were closely associated with high-risk postoperative DVT in TKA patients, and SIRI and AISI values also strongly predicted acute DVT after TKA (10). These inflammatory response indices not only demonstrate sensitivity in patients with traumatic fractures but also enable prediction of DVT risk in other disease populations (31, 34).

However, given that so many inflammatory response indices possess predictive capacity for DVT, does a more specific index exist? A study using the RIETE registry database assessed the predictive value of baseline NLR, PLR, and SII for adverse outcomes at 90 days in patients with acute VTE. None of the three baseline indices were sufficient to predict VTE recurrence in acute VTE patients. Patients with higher baseline NLR values had an increased risk of major bleeding or death, while those with higher SII values only had an increased risk of death (32). Other researchers further analysed that: PLR has some diagnostic and prognostic value for VTE, but further studies are needed to determine its reliability and stability. SII has promising potential value for VTE and warrants further investigation (33). A follow-up study of patients diagnosed with DVT in Turkey found that while the median values of NLR, PLR, and SII were significantly higher in the DVT recurrence group, only SII was identified as a significant and independent predictor of DVT recurrence in multivariate logistic regression analysis (AUC = 0.686, p = 0.001) (14). These findings are supported by studies in other countries (12, 13). We selected SII as a representative index of the inflammatory-inflammatory mechanism for our study; however, given the instability of this index across different studies (35), we believe that other mechanisms are also involved in the formation of DVT.

### Metabolic disorders in the application of DVT

Metabolic syndrome (MetS) is a multifactorial metabolic disorder characterised by a spectrum of cardiovascular metabolic risk factors, including central obesity, hyperglycaemia, elevated blood pressure, and dyslipidaemia. The interaction between these factors not only exacerbates the risk of diabetes but also leads to a prethrombotic state, significantly increasing the risk of cardiovascular diseases, including VTE (37). The 2023 Chinese Guidelines for the Integrated Diagnosis and Treatment of Metabolic Syndrome reported that the prevalence of MetS among Chinese adults aged 20 and above reached 31.1% (36), making MetS an indispensable factor in the pathogenesis of various diseases in the Chinese population. An earlier case-control study on VTE risk conducted at Vienna General Hospital in Austria (40) showed that, without adjusting for confounding factors, the risk of VTE in MetS patients was 2.1 (95% CI [1.2–3.7], p=0.012). After adjusting for established thrombotic risk factors, gender, and age, the risk of VTE increased by 10% (OR = 2.2, 95% CI [1.1–4.3], p=0.020). A recent meta-analysis of 31 case-control and 5 cohort studies further revealed that MetS (OR 1.49; 95% CI 1.29–1.73) and its key components—obesity (OR 2.03; 95% CI 1.74–2.37), hypertension (OR 1.40; 95% CI 1.19–1.64), and diabetes (OR 1.22; 95% CI 1.01–1.48) are important risk factors for VTE (38). The primary mechanism underlying the development of MetS is insulin resistance (IR) caused by reduced peripheral tissue responsiveness to insulin. The TyG index is a new indicator of IR, which only requires a combined analysis of triglycerides (TG) and fasting blood glucose (FBG), making it more widely applicable in clinical practice compared to the traditional hyperinsulinemia-hyperglycaemia clamp test. A prospective cohort study in the Netherlands (39)showed that, after adjusting for traditional cardiovascular risk factors, C-reactive protein (CRP), and endothelial dysfunction markers, the homeostatic model assessment of insulin resistance (HOMA-IR) was associated with an increased risk of VTE (hazard ratio [HR]: 1.38; 95% confidence interval [CI]: 1.09–1.75; P = 0.007). Currently, there is a lack of research on the association between IR and TyG index and the incidence of DVT/VTE in patients with traumatic fractures. A recent study using the TyG index to predict lower extremity venous thrombosis risk in patients with intracerebral haemorrhage (ICH) demonstrated (19)that, in both univariate and multivariate logistic regression analyses, multiple predictive factors including the TyG index were identified as independent risk factors for lower extremity venous thrombosis formation in ICH patients (P < 0.05). It was also noted that reduced insulin binding to insulin receptors in some IR patients may lead to upregulation of the P2Y12 signalling pathway and enhanced platelet activity, promoting platelet adhesion, aggregation, and coagulation processes, ultimately resulting in thrombus formation.

### Chronic metabolic-acute inflammatory antithrombotic mechanism

Our study is the first to comprehensively analyse the incidence of preoperative DVT in trauma fracture patients by integrating the mechanisms of the inflammation-immunity-coagulation axis with the metabolic-coagulation axis. We refer to this composite mechanism as the chronic metabolic-acute inflammatory-coagulation axis. Results from multi-model logistic regression analysis showed that adjusting for confounding variables did not affect the ability of high TyG-SII indices to reduce the risk of DVT and MCVT in patients with traumatic fractures (see **Tables 2**, **3**). Although RCS curves demonstrated a non-linear U-shaped relationship between TyG-SII indices and DVT/MCVT (all P values for non-linear relationships <0.001), the overall incidence remained below the baseline rate. This appears paradoxical, as numerous studies have shown that elevated TyG and SII indices individually are often associated with higher risks of DVT/VTE (10, 11, 19, 30, 31, 34, 39). These findings suggest that while both immune-inflammatory responses and metabolic disorders can independently promote thrombosis, their combined interaction within the TyG-SII composite index may reveal a more complex, potentially antagonistic relationship. We hypothesize that the chronic, low-grade prothrombotic state induced by insulin resistance (reflected by high TyG) and the acute, intense procoagulant burst driven by systemic inflammation (reflected by high SII) may compete for shared components of the coagulation cascade or exert counter-regulatory effects on platelet and endothelial function. This competition could delay the net procogulant process, resulting in the observed lower risk association at higher TyG-SII levels. For example, chronic metabolic dysregulation might modulate the sensitivity of platelets to acute inflammatory stimuli, or alter the cytokine milieu in a way that attenuates the peak thrombotic response following trauma. This hypothesis of pathway competition or buffering offers a plausible explanation for why the composite index behaves differently from its individual components, and aligns with the non-linear, U-shaped association we observed. It also underscores that the two mechanisms, while overlapping, represent distinct pathways that can interact in a non-additive manner following traumatic fractures.

Related studies have shown (19) that under physiological conditions, insulin inhibits platelet aggregation and thrombus formation by enhancing fibrinolysis and inhibiting tissue factor. However, in patients with high insulin resistance (IR), levels of coagulation factors VII, IX, X, and XII are significantly elevated, leading to vascular dysfunction characterised by reduced synthesis of prostaglandins and nitric oxide; accelerating platelet activation while enhancing the coagulation process, thereby inducing a chronic low-grade inflammatory state during thrombus formation (41, 42). This differs from the acute inflammatory response following traumatic fractures, which leads to the systemic release of pro-inflammatory cytokines and the systemic activation of leukocytes and endothelium. In this inflammatory response, the excessive expression of monocytes and neutrophils, along with platelet aggregation, simultaneously activates both the intrinsic and extrinsic coagulation pathways (22). Therefore, we believe that chronic metabolism-induced thrombosis and chronic low-grade inflammatory states, as well as thrombosis caused by immune-inflammatory response processes, both disrupt the anticoagulation-coagulation balance axis and compete for corresponding tissue factors, thrombin, and protein expression during the coagulation-to-thrombosis process. Ultimately, this prolongs the procoagulant processes of the inflammation-immune-coagulation axis and the metabolic-coagulation axis, delays coagulation time, and may even reduce thrombus formation. This perspective is further confirmed by the subgroup analysis results of this study.

Our subgroup analysis of DVT revealed that the risk of DVT decreased with increasing TyG-SII in overweight and diabetic patients. Previous studies have shown (11) that BMI and diabetes, as important components of MetS, promote high-risk VTE episodes, which contradicts our findings. In the subgroup analysis of MCVT, among patients with hypertension and hyperlipidaemia, which are also important features of MetS, the risk of MCVT still decreased with increasing TyG-SII. We propose that when the inflammation-immunity-coagulation axis and the metabolic-coagulation axis act simultaneously in the procoagulant process, chronic metabolic mechanisms dominate, which may be related to the fact that chronic metabolism also induces inflammatory responses. Further mechanistic studies are needed to confirm this. The differences in subgroup analysis between DVT and MCVT may be related to the location of venous thrombosis formation. Most DVT originates from MCVT, so the association between DVT formation and chronic status is stronger (44). Therefore, the BMI index and diabetic status, as core mechanisms of MetS, exhibit more significant interactive effects in the pathogenesis of DVT. As an early manifestation of DVT, MCVT is more susceptible to factors such as hypertension, hyperlipidaemia, age, and gender. Another explanation (43, 44) pertains to the anatomical structure of muscle vessels: their smaller diameter and longer length make them more susceptible to vascular fragility and plaque formation under conditions of hypertension and dyslipidaemia. Additionally, the thrombi formed are smaller in volume compared to those in other lower limb deep veins (such as the femoral vein, popliteal vein, anterior tibial vein, posterior tibial vein, and fibular vein), and upon detachment, they may obstruct other deep veins, leading to DVT. In summary, we should pay close attention to changes in the TyG-SII index, especially as low levels of TyG-SII indicate a higher risk of DVT/MCVT.

### Limitations

Our study has three limitations. First, this is a single-centre retrospective study, and the dataset used mainly comes from the southern population of China, which limits the generalisability of our findings. Second, although this study found evidence of a negative association between TyG-SII and DVT/MCVT, further experimental validation is needed to confirm these associations. Additionally, since our institution does not mandate follow-up lower extremity vascular ultrasound examinations for patients after fracture surgery, we cannot determine changes in DVT/MCVT progression and recurrence before and after fracture surgery in relation to TyG-SII.

## Conclusions

This study revealed a significant negative nonlinear relationship between TyG-SII exposure and the risk of DVT/MCVT in a population with traumatic fractures in southern China. Notably, high BMI and diabetes status had a protective effect on DVT incidence. Young male patients had a lower incidence of MCVT in the presence of hypertension and hyperlipidaemia. This may reveal that metabolic disorders play a dominant role in thrombosis formation.

## Data Availability

The raw data supporting the conclusions of this article will be made available by the authors, without undue reservation.
